# Editorial: Barriers to Promoting Physical Activity in Low- and Middle-Income Countries: Alignment With the Sustainable Development Goals

**DOI:** 10.3389/fpubh.2022.943428

**Published:** 2022-06-09

**Authors:** Wei Liu

**Affiliations:** ^1^Key Research Base of Humanities and Social Sciences in Colleges and Universities in Anhui – Quality Education Research Center for College Students of Anhui Xinhua University, Hefei, China; ^2^Department of Physical Education, Anhui Vocational and Technical College of Sports, Hefei, China

**Keywords:** physical activity, snow and ice participation, execution effect, class identity, sustainable development goals

One of the Sustainable Development Goals supported by the United Nation is good health and wellbeing. A key component is to reduce one-third of premature mortality by increasing physical activity levels. To achieve this target, we need research on the low and middle-income countries (LMIC) to support evidence-informed policy-making, including population-based policies that encourage public participation and target health, and education to motivate the public for physical activity.

However, there are still many social barriers to promoting physical activity in low-income and middle-income countries, including the participation of special groups, insufficient resources in ice and snow sports, and a lack of facilities for rural residents, which should become the focus of researchers. More in-depth research can help people in LMIC benefit from physical activities.

It is an interdisciplinary study involving contents in medicine, sociology, and sports. For example, for pregnant women in South Africa, the lack of prenatal physical activity is reportedly due to insufficient relevant information, tiredness, work commitment, discomfort, lack of time, and low energy (Okafor and Goon). It is suggested that the government should adjust the intervention measures to solve their uniquely perceived barriers (Okafor and Goon). In response to the 2022 Winter Olympic Games held in Beijing, the Chinese government has issued policies such as “South exhibition, West Expansion, and East Expansion” of ice and snow sports to promote and encourage the participation of the broad masses of the people. However, in fact, there are economic difficulties for middle-income and low-income groups in participating in ice and snow sports. The research on the subject of this study suggests that it is necessary to give help to middle-income and low-income groups to increase their participation in ice and snow sports to reduce the inequalities in physical activity between them and the cultural elite groups (Li and Liu). Similarly, studies have found that most rural residents in the Zhejiang Province of eastern China had longer daily sedentary time (ST) and less physical activity (LPA). This was predominant in men, young people, highly educated people, unmarried people, and middle to high-income people. Health education programs should be targeted toward specific population groups to decrease the ST and increase PA (Wang et al.). Another topic that has attracted much attention is the evaluation of the relationship between Chinese urban and rural residents' participation in sports activities from the perspective of class identity. The study found that physical activity is positively correlated with the subjective class identity of urban and rural residents, and confirmed that subjective class identity can significantly improve residents' sports activities. Therefore, national and local governments should promote equality in sports by giving financial support to increase public facilities, and enhance participation in sports activities by improving subjective class identity (Yang et al.). In the last article on this Research Topic, it was found that the physical activities of Chinese college students have a positive role in promoting the physical activities of their parents (Liu et al.). It shows the dynamic role of individuals in the process of socialization and the two-way nature of socialization. With the transformation from traditional society to modern society, promoting college students' sports activities can increase parents' sports activities and improve the level of social sports activities.

Through the topic of this research, we can see that middle-income and low-income countries and regions need to pay more attention to public health issues from the social level in promoting physical activities. Social stratification, social mobility, and social inequality are the key factors leading to the lack of availability of physical activities in middle-income and low-income countries and regions. In summary, this research theme has taken an important step toward further promoting the development of physical activity worldwide, but there is still a long way to go. At this level, social factors are one of the main factors to be considered.

## Author Contributions

WL drafted the work.

## Conflict of Interest

The author declares that the research was conducted in the absence of any commercial or financial relationships that could be construed as a potential conflict of interest.

## Publisher's Note

All claims expressed in this article are solely those of the authors and do not necessarily represent those of their affiliated organizations, or those of the publisher, the editors and the reviewers. Any product that may be evaluated in this article, or claim that may be made by its manufacturer, is not guaranteed or endorsed by the publisher.

